# Evaluation of an aminobisphosphonate (alendronate) in the management of periodontal osseous defects

**DOI:** 10.4103/0972-124X.65438

**Published:** 2010

**Authors:** H. R. Veena, Deepak Prasad

**Affiliations:** *Assistant Professor, Department of Periodontics, KLE Institute of Dental Sciences and Research Centre, Yeshwanthpur Suburb, Bangalore-560 022, Karnataka State, India*; 1 *Professor and Head, Department of Periodontics, Farooquia Dental College and Hospital, Umer Khayyam Road, Tilak Nagar, Mysore – 570 021, Karnataka State, India*

**Keywords:** Alendronate/therapeutic use, alveolar bone loss, bisphosphonates/therapeutic use, bone resorption, osteoclasts, surgical flaps

## Abstract

**Background and Objectives:**

Alendronate, an aminobisphosphonate, is capable of inhibiting periodontitis associated osteoclastic activity and hence is effective in protecting the alveolar bone in periodontitis. In the present study, we explored the efficacy of local delivery of alendronate on the alveolar bone following mucoperiosteal flap surgery. This is the first study to use polymer impregnated gel based delivery of alendronate.

**Materials and Methods:**

A total of 15 patients with chronic periodontitis in the age group of 35 - 55 years, of both sexes, with pocket depth of ≥ 5mm and radiographic evidence of identical osseous defects in the mandibular molar region bilaterally were included in this prospective study. A gel based drug delivery system of Alendronate was formulated. Following surgical flap debridement, 0.1 ml alendronate gel and 0.1 ml placebo gel was placed at the experimental and control sites respectively. Clinical and radiographic parameters were recorded at baseline, three months and six months post surgery.

**Results:**

Alendronate was more effective in improving clinical and radiographic parameters compared to placebo.

**Interpretation and Conclusion:**

Alendronate is effective in the management of periodontitis associated bone loss. Gel based local delivery of the drug addresses the critical concern of exposing the patient to adverse effects of systemic administration.

## INTRODUCTION

Bisphosphonates are carbon substituted pyrophosphate analogs that are potent inhibitors of bone resorption and have been effectively used to treat metabolic bone diseases in humans, which include Paget’s disease, hypercalcaemia of malignancy, osteoporosis and estrogen deficiency.[1] Like pyrophosphate, they bind to the hydroxyapatite crystals of bone and prevent their dissolution.[2]

Alendronate (4-amino 1-hydroxybutylidine bisphosphonate), a novel bisphosphonate is a very potent inhibitor of bone resorption (six to 10 times more potent than pamidronate and up to 1000 times more potent than etidronate) and therefore used in low concentrations than etidronate and clodronate. Furthermore, no inhibition of mineralization has been described at doses used pharmacologically. The net effect of alendronate on bone formation might be explained by its inhibition of osteoclasts, thus affecting bone maturation and remodeling.[3] Once taken up by bone, alendronate has a prolonged skeletal retention (half-life up to several years) and significant amounts can be released in the resorptive process which may in turn provide protection to the alveolar bone.[2]

It has been known for a long time that systemically administered bisphosphonates can induce gastrointestinal disturbances such as esophagitis, erosions, and ulcerations.[4] Local drug delivery avoids most of these problems, by limiting the drug to the target site (site specific approach) with little or no systemic uptake. Also, the local concentration achieved can be much higher (100 folds) than is possible via systemic route.[5]

### Aims and objectives

1. To evaluate clinically and radiographically, the efficacy of an aminobisphosphonate (alendronate) used locally in the management of periodontal osseous defects.
2. To evaluate the periodontal tissue response to alendronate by recording clinical parameters (Gingival index, probing pocket depth and clinical attachment level) and radiographic parameters.

## MATERIALS AND METHODS

A total of 30 sites were selected from 15 patients with chronic periodontitis in the age group of 35 to 55 years of both sexes, with no history of metabolic disorders involving bone resorption (eg: Paget’s disease, osteoporosis, malignancies and estrogen deficiencies) and pocket depth of ≥5 mm with radiographic evidence of identical osseous defects in the mandibular molar region bilaterally. An informed consent was obtained from all the patients prior to their enrollment for the study.

All patients received oral hygiene instructions and complete full mouth scaling and root planing prior to surgical treatment. A split mouth design was employed and the clinical and radiographic parameters were recorded at baseline, three and six months post surgery.

**Figure 1:**
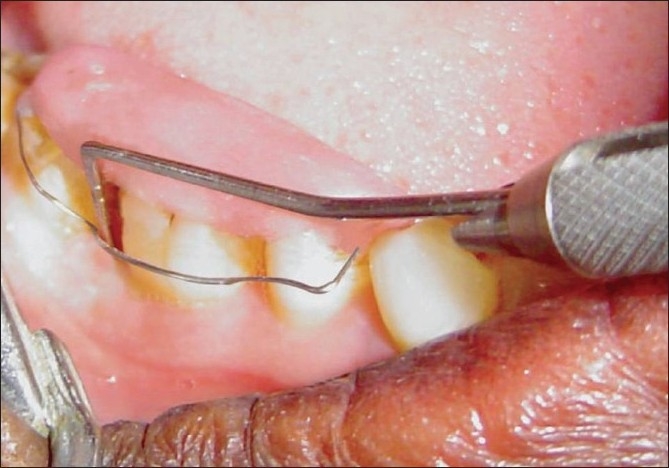
Pocket depth measurement

In the present study, the problems of clinical periodontal probing were minimized by using a constant pressure probe (Brockprobe™)[6] and using customized acrylic stents with guiding grooves for reproducible probing sites and directions [Figure 1].

### Brockprobe™: (U.S Patent # 5,000,683, Brockport Industries, Hackettstown, NJ)

Brockprobe is a second generation pressure sensitive probe with William’s markings (1, 2, 3, 5, 7, 8, 9,10 mm). The probe exerts a standardized pressure of 20 grams.[7] The probe is gently inserted into the sulcus/pocket until the shaft flexes and meets the top stop. At this point (20 grams), the proper probing pressure has been reached and the depth reading is noted.[6] Sterilization of the probe can be achieved by autoclave, chemiclave or dry heat.

Intra oral periapical radiograph of each defect site was exposed using Long Cone Paralleling technique. Kodak Ekta speed films were used as it reduced the radiation dose by a factor of 8 compared to D speed film. Exposures were made at 70 KVp, 8 ma for 0.6 seconds as higher kilovoltage X-rays are less likely to be absorbed in the tissues and result in lower contrast radiograph that show more shades of gray.[8] The focus to film distance was 20 cms and the total filtration was 2 mm of aluminum.

The mandibular molar region was the selected site for the study as the film could be placed parallel and closer to the tooth and the thick bony trabeculae are aligned horizontally and closer to each other.[9] Periapical films are susceptible to operator errors in the maxillary molar region due to the overlapping of the palatal root over the osseous defect and the anatomy of the palatal vault.[8] The radiographs were taken by the same radiologist throughout the study to minimize discrepancies.

All the radiographs were scanned and digitized using HP-transparency scanner. The digitized images were displayed on the monitor at 5× magnification. Linear measurements were made on the digitized images using Adobe photo shop 5.5 computer software.

**Figure 2:**
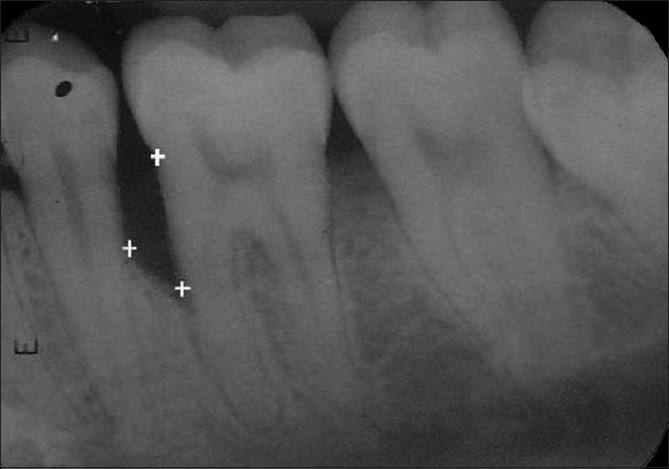
Method of recording linear radiographic measurements

The following landmarks were marked on the digitized image of the radiograph [Figure 2]

- Cementoenamel junction(CEJ)
- Alveolar crest (AC)
- Base of the defect (BD)

Formulation of gel based drug delivery system of alendronate/ placebo: The gel containing alendronate sodium for local delivery was prepared using pharma grade materials. The drug was formulated in a gel form for the ease of placement and retention at the target site following placement.

About 200 mg of alendronate sodium was dissolved in 100 ml of distilled water. To this, 200 mg of Carbopol 934 P was added to get a concentration of 1 %. The mixture was stirred gradually and Carbopol was allowed to soak for 2 hours. 0.5 ml of triethanolamine was added to the gel and finally 30 mg of methyl paraben and 10 mg of Propyl paraben were dissolved in 2 ml of ethanol and added to the preparation. The gel formulation was sterilized by autoclaving for 30 minutes at 121°C. The sterility of the formulation was tested using culture media for both aerobic and anaerobic bacteria. The *in vitro* release of the drug studied using dialysis membrane showed 100 % drug release in 14 hours.

As a placebo, alendronate-free gel with identical basic composition was prepared.

The formulations were transferred to 2 ml syringes under sterile conditions and dispensed for clinical study.

### Surgical procedure

**Figure 3:**
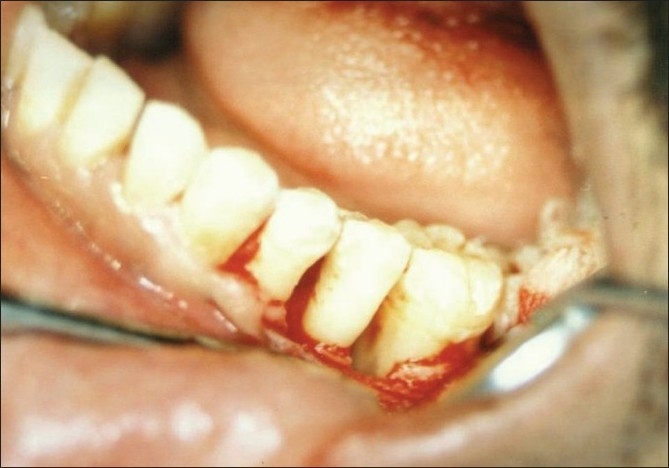
Osseous defect

The selected sites were randomly assigned as either control or experimental site. After adequate anesthesia of the surgical site, a full thickness mucoperiosteal flap was reflected and the osseous defect was exposed [Figure 3]. A thorough surgical degranulation of the infected tissue was done and the surgical site was thoroughly irrigated with saline. In the control site, 0.1 ml of the placebo gel was delivered to the osseous defect.

**Figure 4:**
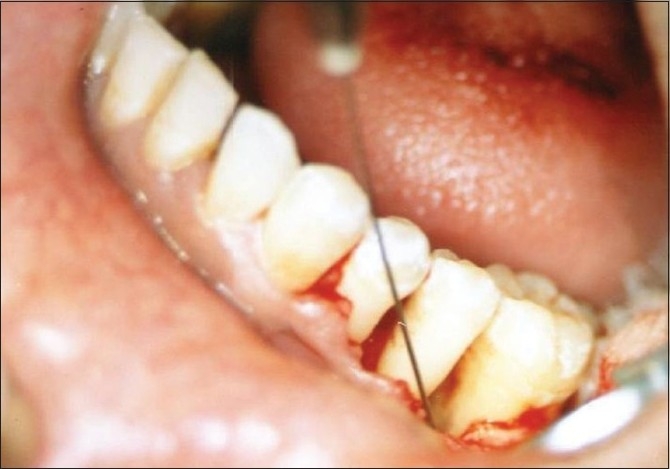
Placement of alendronate gel

In the experimental site, 0.1 ml (200 μ gms)[1] of alendronate gel was delivered to the osseous defect [Figure 4]. The mucoperiosteal flap was repositioned and secured in place using black braided (4-0) silk. The surgical site was protected with a non-eugenol periodontal dressing.

## RESULTS

The statistical techniques employed for the analysis of data obtained were, ANOVA-repeated measures, independent sample ‘*t*’ test, paired sample ‘*t*’ test.

**Graph 1:**
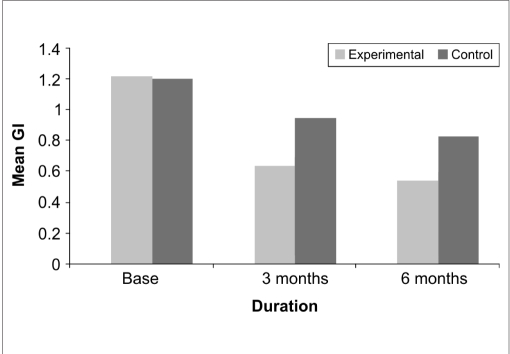
Postoperative changes in gingival index

With respect to Gingival index, at three months post treatment, a mean value of 0.9500±0.1615 and 0.6400±0.3376 at the control and experimental sites respectively and ‘*t*’ value of -3.208 indicated a statistically significant (*P*<0.003) difference. At the end of six months, the control and experimental sites revealed a mean value of 0.8333±0.1113 and 0.5467±0.2825 respectively, with a ‘*t*’ value of -3.657 indicating a statistically significant (*P*<0.001) difference [Table 1, Graph 1]

**Table 1:** Comparison of baseline and postoperative changes in gingival index scores between the two sites

Source	Sum of squares	df	Mean square	F	Sig.
Change	4.555	2	2.278	77.460	0.000
Change * site	0.515	2	0.258	8.759	0.000
Error (CHANGE)	1.647	56	0.029		

* -with respect to

**Graph 2:**
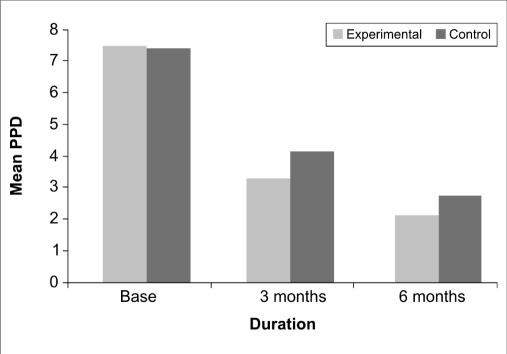
Postoperative changes in probing pocket depth

At baseline, a mean pocket depth of 7.40±1.18 mm and 7.47±1.13 mm at the control and experimental sites respectively with a ‘*t*’ value of 0.158 indicated a non-significant (*P*<0.876) difference between the two sites. At three months post treatment, the values showed a mean probing pocket depth of 4.13±0.64 mm and 3.27±0.46 mm at the control and experimental sites respectively with a ‘*t*’ value of -4.266 indicating a statistically highly significant (*P*<0.000) difference. At the end of 6 months, The control and the experimental sites revealed a mean score of 2.73±0.46 mm and 2.13±0.35 mm respectively with a ‘*t*’ value of -4.025 indicating a statistically highly significant (*P*<0.000) difference [Table 2, Graph 2].

**Table 2:** Comparison of baseline and postoperative changes in probing pocket depth measurement between the two sites

Source	Type III Sum of squares	df	Mean square	F	Sig.
Change	405.422	2	202.711	399.087	0.000
Change * site	3.467	2	1.733	3.412	0.040
Error (CHANGE)	28.444	43.278	0.657		

* -with respect to

**Graph 3:**
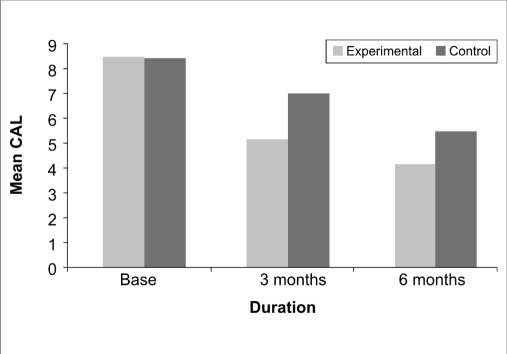
Postoperative changes in clinical attachment level

With respect to clinical attachment level, at baseline, a mean value of 8.4±1.1832 mm and 8.4667±1.1255 mm at the control and experimental sites respectively with a ‘*t*’ value of 0.158 indicated a non-significant (*P*<0.876) difference between the two sites. At three months post treatment, the mean values of 7.0±1.0000 mm and 5.1333±0.5164 mm at the control and experimental sites respectively and a ‘*t*’ value of -6.424 indicated a statistically highly significant (*P*<0.000) difference. At the end of six months, the control and experimental sites revealed a mean value of 5.4667±0.9904 mm and 4.1333±0.8338 mm respectively with a ‘*t*’ value of -3.989 indicating a statistically highly significant (*P*<0.000) difference [Table 3, Graph 3].

**Table 3:** Comparison of postoperative changes in clinical attachment level measurement between the two sites

Source	Type III Sum of squares	df	Mean square	F	Sig.
Change	204.067	2	102.033	119.927	0.000
Change * site	14.956	2	7.478	8.789	0.000
Error (CHANGE)	47.644	56	0.851		

* -with respect to

**Figure 5:**
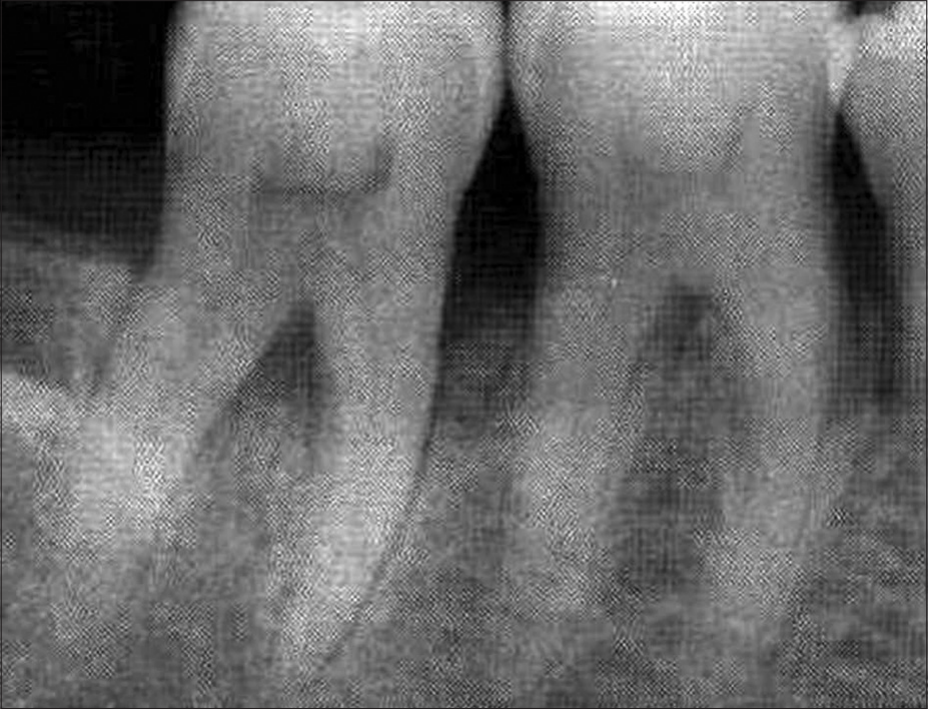
Radiograph of experimental site at baseline

**Figure 6:**
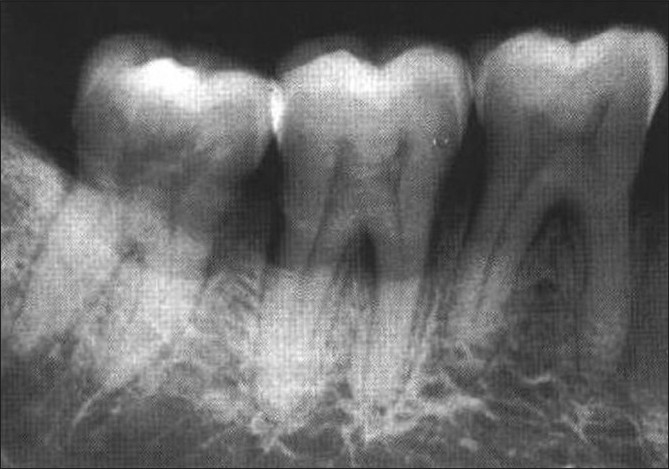
Radiograph of experimental site at 3 months

**Figure 7:**
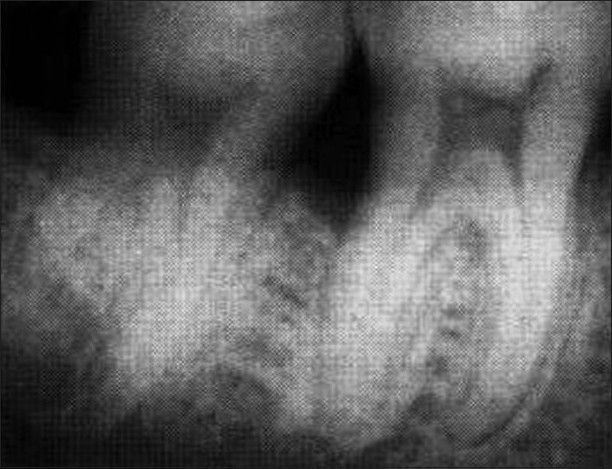
Radiograph of experimental site at 6 months

**Graph 4:**
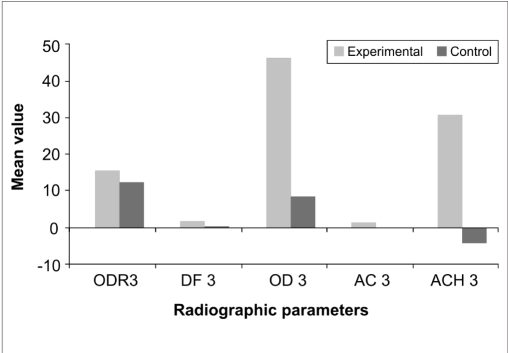
Mean changes in radiographic parameters between the two sites at 3 months

**Graph 5:**
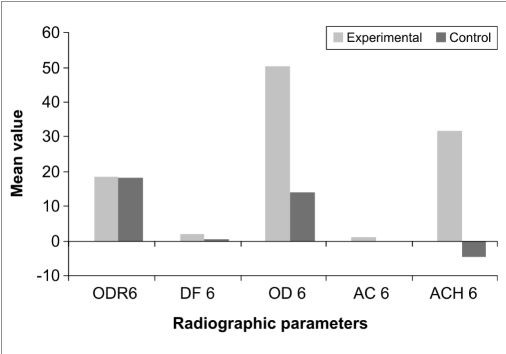
Mean changes in radiographic parameters between the two sites at 6 months

There was a statistically non-significant difference in the percentage of original defect resolved at the end of three months (*P*<0.177) and six months (*P*<0.902). With respect to the amount of defect fill, there was a statistically significant difference at the end of three months (*P*<0.000) and six months (*P*<0.000) with the experimental site showing a better result than control. There was a statistically significant difference in the percentage fill of original defect at the end of three months (*P*<0.000) and six months (*P*<0.000) with the experimental site showing a better result than control. A statistically significant change in alveolar crestal height was seen at the end of three months (*P*<0.000) and six months (*P*<0.000) with the experimental site showing alveolar crestal height gain and the control site showing alveolar crestal resorption [Tables 4 and 5, Figures 5–7, Graphs 4 and 5].

**Table 4:** Comparison of radiographic parameters between the two sites 3 months post surgery

Radiographic parameter	t	df	Sig. (2-tailed)	Mean difference
Percentage of original defect resolved (ODR3)	1.385	28	0.177	3.2110
Amount of defect fill (DF3)	14.433	28	0.000	1.5953
Percentage fill of original defect (OD3)	9.840	28	0.000	37.7457
Change in alveolar crest (AC3)	15.948	28	0.000	1.4127
Percentage change in alveolar crest height (ACH3)	10.995	28	0.000	34.9493

Independent sample ‘*t*’ test

**Table 5:** Comparison of radiographic parameters between the two sites 6 months post surgery

Radiographic parameter	t	df	Sig.(2-tailed)	Mean difference
Percentage of original defect resolved (ODR6)	0.124	28	0.902	0.3768
Amount of defect fill (DF6)	13.073	28	0.000	1.8373
Percentage fill of original defect (OD6)	9.071	28	0.000	42.7678
Change in alveolar crest (AC6)	15.429	28	0.000	1.7280
Percentage change in alveolar crest height (ACH6)	11.881	28	0.000	42.3928

Independent sample ‘*t*’ test

## DISCUSSION

This is the first study to report the local delivery of alendronate as a gel based polymer impregnated drug (pubmed search). It is evident from the mean values that proportionately more decrease in Gingival index was observed in the experimental group compared to control group.

This finding was in accordance with the findings of Rocha M *et al*,[10] who found an improvement in gingival index in both the Alendronate and placebo group, with the Alendronate group showing better results. With respect to the probing pocket depth, at the three months and six months post treatment follow-up, the experimental site showed a highly significant reduction in probing pocket depth compared to control.

Similar results were obtained by Rocha M *et al*,[10] who reported a trend towards decreased pocket depth after treatment with alendronate compared to control. Brunsvold MA *et al*,[11] in a monkey model of periodontitis treated with Alendronate did not observe an effect on probing depth measurement. This lack of effect of Alendronate in this work may be explained by the short duration of systemic administration of the drug.

The postoperative percentage of defect fill was greater in the experimental site (59.27%) compared to the control site (16.5%). These findings were in accordance with the findings of Meraw SJ *et al*,[3] who demonstrated by secondary fluorescence examination that locally applied Alendronate was influential in increasing bone formation rates, Reddy MS *et al*,[12] who over a six month’s study demonstrated by histomorphometric analysis that Alendronate administration represented a drug induced gain in bone density and improved bone mass when used to treat periodontal defects. Brunsvold MA *et al*,[11] also demonstrated that Alendronate clearly reduced loss in bone density as measured radiographically.

The percentage of original defect resolved showed a non-significant change (*P*<0.902) over a 6 month duration between the experimental and control sites. A significant improvement in alveolar crest height at the experimental site over a period of six months was found. An intriguing aspect in the present study was that the control site showed alveolar crest resorption. This data was in accordance with the findings of Binderman I *et al*,[1] Kaynak D *et al*,[13] Yaffe A *et al*,[14] which demonstrated that Alendronate was effective in reducing alveolar bone loss when delivered at the surgical site.

Similar findings were observed by Rocha M *et al*,[11] who demonstrated that Alendronate administration increased alveolar bone height and decreased the distance from the cemento-enamel-junction to the alveolar bone. This change was significant when compared to the placebo group in which the distance increased.

From a clinical point of view, Alendronate was very well tolerated with no overt side effects. The post operative healing was uneventful. This was in accordance with the findings of Brunsvold MA *et al*,[12] Weinreb M *et al*,[15] Meraw SJ *et al*[3] and Rocha M *et al*.[11]

Osteonecrosis of the bone, especially of the jaw (ONJ), has been recently reported with the use of oral bisphosphonates following the treatment for osteoporosis. To date, fewer than 50 cases have been reported worldwide.[16] This number is very less compared to the extensive use of bisphosphonates in osteoporosis. Masoodi NA reported that ONJ is more frequently associated with high doses of intravenous drug.[17] Myelomas have also been reported with oral bisphosphonates. However, we used the drug at very low concentrations and dosage locally. We did not note any such complications in the study period. We recommend that physicians be aware of such an occurrence and should discuss the same with patients. Long term studies would answer the incidence of such a disease after the local drug delivery.

## CONCLUSIONS

1. Both treatment modalities improved the overall periodontal status though the best results were obtained in the site treated with Alendronate.
2. The local delivery of Alendronate introduced in this study as a gel based polymer impregnated drug was found to be safe with no impact on the wound healing and had no local adverse events.

### Clinical / research implications

1. The use of bisphosphonates as an adjunct to mechanical and surgical regenerative treatment approaches may be the future potential applications of periodontal therapy
2. It may be interesting to use them with anti-inflammatory drugs to manage more conceivable results of periodontal therapy.
3. For surgical approaches in dentistry where bone graft materials and/or dental implants are needed, the use of bisphosphonates may achieve a new dimension for periodontal therapy.
